# TFCP2 Overcomes Senescence by Cooperating With SREBP2 to Activate Cholesterol Synthesis in Pancreatic Cancer

**DOI:** 10.3389/fonc.2021.724437

**Published:** 2021-11-04

**Authors:** Dexiang Zhang, Pinxiang Lu, Kaihua Zhu, Haixia Wu, Yuedi Dai

**Affiliations:** ^1^ General Surgery Department, Central Hospital of Xuhui District, Shanghai, China; ^2^ Department of Medical Oncology, Fudan University Shanghai Cancer Center, Minhang Branch, Shanghai, China

**Keywords:** pancreatic cancer, cell senescence, cholesterol synthesis, TFCP2, SREBP2

## Abstract

KRAS mutation is very common in pancreatic cancer. How pancreatic cancer cells overcome oncogene-induced senescence is not fully understood. Our previous studies showed that up-regulation of TFCP2 (transcription factor CP2) in pancreatic cancer promoted the growth and metastasis of pancreatic cancer cells. However, whether TFCP2 plays an important role in pancreatic cancer cell senescence is not clear. In this study, we found upregulation of TFCP2 expression in pancreatic cancer was associated with KRAS mutation. Overexpression of TFCP2 inhibited cell senescence. Knockdown of TFCP2 promoted cell senescence. Mechanistically, the interaction between TFCP2 and SREBP2 (sterol regulatory element binding transcription factor 2) synergistically activated the expression of HMGCR, a rate-limiting enzyme in cholesterol synthesis, and statins could reverse the inhibitory effect of TFCP2 on senescence. In conclusion, our study reveals a new mechanism underlying the TFCP2 regulation of pancreatic cancer cell senescence, providing a new target for the treatment of pancreatic cancer.

## Introduction

Pancreatic cancer is one of the most common malignancies (1). Although great progress in diagnosis and treatment are made for pancreatic cancer, its five-year survival rate has barely improved (2). Metabolic reprogramming is a basic feature of tumor cells (3). Intensive research into the mechanisms underlying the metabolic reprogramming would provide novel insight into the treatment of pancreatic cancer.

The transcription factors of TFCP2/Grainyhead family are divided into two different subfamilies: one includes GRHL1, GRHL2 and GRHL3, and the other is composed of TFCP2, TFCP2L1 and UBP1 (4). TFCP2 plays important roles in multiple biological events, such as tumor progression (5), stem cell maintenance, angiogenesis, senescence (6), etc. TFCP2 is a proto-oncogene for hepatocellular carcinoma and breast cancer, and may drive the progression of cervical cancer and colorectal cancer (6). TFCP2 may also serve as a tumor suppressor in melanoma (7). In the previous studies, we found that high expression of TFCP2 in pancreatic cancer promoted pancreatic cancer cell growth and colony formation and activated the β-catenin/TCF signaling pathway (8). Expression of TFCP2 is regulated by H-ras, microRNA and the like (9). CCT3 prevents the ubiquitination of TFCP2 and YAP by inhibiting ubiquitinating enzyme PCBP2, thereby prolonging their half-lives (10).

Cell senescence is generally linked to the inhibition of tumorigenesis (11–13). Some oncogenes can induce cell senescence (14). How tumor cells overcome the senescence is not fully clarified. P53, P21, P27, Rb and the like are important regulators of cell senescence (12). Environmental factors (such as rays and nutritional deficiency) can induce cell senescence (15, 16). SREBP2 is an important transcription factor that regulates intracellular cholesterol synthesis (17). Processing and consequently nuclear translocation of SREBP2 entails the participation of three accessory proteins, SREBP2 cleavage activating protein (SCAP), site 1 protease (S1P), and site 2 protease (S2P) (18). Upon stimulation by various factors, SCAP escorts SREBP2 to the Golgi apparatus where they are clipped, sequentially, by S1P and S2P. The liberated/mature SREBP2, the N-terminus of SREBP2, designated as SREBP2N, moves into the nucleus functioning as a pro-lipogenic transcription factor to regulate lipid homeostasis and disorder (18, 19). Rate-limiting enzyme HMGCR for *de novo* synthesis of cholesterol is an SREBP2 downstream target gene (20). It has been reported that treatment of glioma cells with HMGCR inhibitor statins can induce cell senescence (21). Currently, studies show that TFCP2 knockdown can induce the mitotic delay and senescence of Hela cells (22), but the mechanism is unclear.

In this study, we investigated the effect of TFCP2 expression on the senescence of pancreatic cancer, and explored the underlying molecular mechanism.

## Materials and Methods

### Clinical Samples

Human pancreatic cancer samples were obtained from Xuhui Central Hospital of Fudan University with the informed consent from the patients. This study was approved by the Ethics Committee, Xuhui Central Hospital of Fudan University. All tissues obtained were soaked in 4% paraformaldehyde solution, dehydrated, dewaxed, and paraffin-embedded.

### Cell Culture and Transfection

Human pancreatic normal cell lines HPDE6C7 and HPNE, cancer cells [BXPC3, HsT766T, HPAC (G12D), CFPAC (G12V) and MIAPaCa2 (G12C)] were purchased from Cell Bank, Chinese Academy of Sciences. These cells were cultured in 10 cm Petri dishes with DMEM [supplemented with 10% serum (GIBCO) and antibiotics (GIBCO)]. Cells were placed in an incubator with 5% CO_2._

The coding sequences of TFCP2, Kras^G12D^ and 1-1455bp of SREBP2 (SREBP2N, the N-terminus of SREBP2) were inserted into the pLVX-IRES-puro vector. The shRNAs targeting TFCP2 and HMGCR were designed with the help of the Sigma website, and cloned into the pLKO.1-puro vector. The shRNA sequences were: shTFCP2 1#, 5’-aatcaaggacagtcttatgaa-3’; shTFCP2 2#, 5’-aatatactgagcacttacac-3’; shHMGCR 1#, 5’- aagaattgacaggcttgaat-3’; shHMGCR 2#, 5’- aacctgaaattgaacttcc ca-3’. The lentivirus was packaged in 293T cells, with psPAX2 and pMD2.G as the packaging vectors. After being concentrated at PEG8000, the collected virus solution was centrifuged for 1h (4°C, 1600g). After removing the supernatant, the virus was dissolved in 2ml of DMEM. The cells were seeded into a 6-well plate at a density of 50%-60%. The next day, 400ul of lentivirus was added to the cells and placed in a constant temperature incubator for incubation overnight. Two days later, the cells were cultured with puromycin (1 mg/mL) for 4 days. Then, the resistant cells were pooled, and western blot was used to detect the expression of TFCP2.

### Immunohistochemistry (IHC)

A paraffin-embedded tissue was sectioned into 5 μm-thick sections. The sections were soaked in xylene solution for 10 min and then deparaffinized. Then the sections were hydrated with absolute alcohol, 95% ethanol, 85% ethanol, 75% ethanol, and double distilled water successively. The sections were soaked in sodium citrate solution (pH 6.0) heated to 100°C and boiled for 20 min for antigen retrieval. After cooling to room temperature, the sections were treated with 0.3% H_2_O_2_ for 30 min to remove endogenous peroxidase. The sections were blocked with 5% BSA for 1 h at room temperature and then incubated with primary antibody (anti-TFCP2, Sigma, HPA070247, 1:100) diluent overnight, followed by washing with PBS thrice for 5 min each. The sections were incubated with secondary antibody for 1 h at room temperature, followed by washing with PBS thrice for 5 min each. The sections were stained with DAB for 2 min and flushed with running water for 1 min to terminate the chromogenic reaction. Then, the sections were stained with hematoxylin for 3 min, and flushed with running water. The sections were treated with 1% hydrochloric acid in ethanol for 5 s, blue-stained back with running water, dried, sealed, and photographed. Both the staining intensity and protein expression level were automatically scored by the Vectra 2 system. The protein levels of TFCP2 were evaluated based on the percentage of positive cells and staining intensity (0, negative; 1+, weak; 2+, moderate; 3+, strong) using the H score. The H score is a product of the percentage of cells in each intensity category (0, 1+, 2+ and 3+). H-score was calculated by the software using the following formula: H-score=3* (% of 3+ cells) + 2* (% of 2+ cells) + 1*(% of 1+ cells).

### Western Blot (WB)

The cell culture medium was discarded, and cells were washed with PBS twice. Cells were lysed with RIPA Lysis Buffer (containing protease inhibitor and phosphatase inhibitor) for 20 min, and centrifuged for 20 min at 12,000 rpm and 4°C. The supernatant was aspirated, protein concentration was determined by Bradford assay, and SDS-PAGE was performed. After SDS-PAGE, the protein was transferred to a PVDF membrane. At room temperature, the PVDF membrane was blocked with 5% skim milk for 1 h. Then, the membrane and the primary antibody diluent were blocked overnight. The next day, the membrane was washed with TBST thrice for 10 min each. The secondary antibody was incubated with the membrane for 1 h at room temperature. The membrane was washed with TBST thrice for 10 min each. Then the assay was performed by ECL. The primary antibodies used in this experiment were as follows: TFCP2 (proteintech, 15203-1-AP, 1:1000), tubulin (Santa Cruz Biotechnolog, sc-5286, 1:4000), Flag (Sigma, F9291; 1:3,000), HA(Sigma, H3663, 1:2000), GAPDH (proteintech, 60004-1-Ig, 1:5000), GST (proteintech, 10000-0-AP, 1:3000), P16 (proteintech, 10883-1-AP, 1:1000), P27 (proteintech, 25614-1-AP, 1:1000), P21 (proteintech, 10355-1-AP, 1:1000), SREBP2 Full length (Abcam, ab112046, 1:1000), SREBP2 N-terminal (Abcam, ab30682, 1:1000), HMGCR (Abcam, ab174830, 1:1000).

### Sphere Formation

The cells (1,000 cells for each well) were suspended in the media containing DMEM/F12 supplemented with 2 mM L-glutamine, 100 U/ml penicillin, 100 U/ml streptomycin, 20 ng/ml recombinant human epidermal growth factor (EGF; Sigma), 10 ng/ml recombinant human basic fibroblast growth factor (bFGF; R&D Systems) and 1x B27 supplement. Then, cells were seeded into ultra low-attachment 6-well plates (Corning) and placed in the incubator for 5-10 days. At the end of the assay, the spheres were photographed and counted.

### β-Gal Staining

Cell senescence was detected by Senescence β-Galactosidase Staining Kit (Beyotime). The cell culture medium was aspirated and cells were washed with PBS once and fixed with 1 mL of β-galactosidase Fixative Solution for 15 min at room temperature. The fixative solution was aspirated, and cells were washed with PBS thrice for 3 min each. PBS was aspirated, and 1 mL of Staining Solution was added to each well, followed by incubation overnight at 37°C. Cells were photographed and counted under a common optical microscope.

### Co-Immunoprecipitation (Co-IP)

Cells were lysed with RIPA Lysis Buffer (containing protease inhibitor and phosphatase inhibitor) for 20 min and centrifuged for 20 min (12,000 rpm, 4°C). The primary antibody was added to the supernatant and incubated on a shaker overnight at 4°C. The next day, 100 μL of Protein A/G Agarose Beads (Sigma) were added, followed by incubation for 4 more hours. The beads were washed with RIPA Lysis Buffer thrice for 10 min each. After the Lysis Buffer was removed, 40 μL Loading Buffer was added, followed by heating for 5 min at 100°C. The supernatant was used for western blot.

### GST Pull-Down

Cells were lysed with RIPA Lysis Buffer (containing protease inhibitor and phosphatase inhibitor) for 20 min, and centrifuged for 20 min (12,000 rpm, 4°C). The supernatant was mixed with 10 μg of GST-SREBP2N fusion protein and incubated on a shaker overnight at 4°C. The next day, 100 μL of Sepharose 4B agarose beads (GE healthcare) were added, followed by incubation for 4 hours. The beads were washed with RIPA Lysis Buffer thrice for 10 min each. The RIPA Lysis Buffer was removed. 40 μL of Loading Buffer was added, followed by heating for 5 min at 100°C. The supernatant was used for western blot.

### qPCR

RNA was collected and extracted with Trizol, and transcribed to cDNA using Promega Reverse Transcription System. qPCR was conducted using SYBR Green Premix (Takara) with the Stratagene MX3000P program. The reaction program was as follows: 95°C for 2 min; 45 cycles of 95°C for 15 s, 55°C for 15 s, and 68°C for 30 s. Subsequently, melt curves were plotted at 65-95°C. The HMGCR forward primer was 5’-TGATTGACCTTTCCAGAGCAAG-3’; the HMGCR reverse primer was 5’-CTAAAATTGCCATTCCACGAGC-3’. The GAPDH (internal control) forward primer was 5’-GGAGCGAGATCCCTCCAAAAT-3’; the GAPDH (internal control) reverse primer was 5’-GGCTGTTGTCATACTTCTCATGG-3’.

### Chromatin Immunoprecipitation Assay

The cells were plated into a 10 cm dish. When the confluence reached 90%, the cells were cross-linked with 1% paraformaldehyde for 10min, and incubated with 125 mM glycine for 3min at room temperature. Then, the cells were washed twice in cold PBS, and collected using 2ml of DTT solution (100 mM pH 9.5 Tris-HCl, 10 mM DTT) for 10min of incubation at room temperature. Finally, the cells were centrifuged (4°C, 5000g) for 5min. The cell sediment was resuspended in 150μL of SDS solution (50 mM Tris-HCl at pH 8.0, 2 mM EDTA and 1% SDS) containing protease and a phosphatase inhibitor. The DNA fragments were cut into smaller ones with an average of about 500bp using ultrasound with a power of 12%, and examined using the Expressshearing Kit (Active motif, 53008). The binding of SREBP2 to the HMGCR promoter was analyzed with qPCR. The PCR primer was designed as follows: F: 5’- CAAGGTCGGGAGTGATGATG -3’, R: 5’- TTCCTGTGCGAACCTTAC -3’.

## Results

### TFCP2 Was Up-Regulated in Pancreatic Cancer

**Figure 1:**
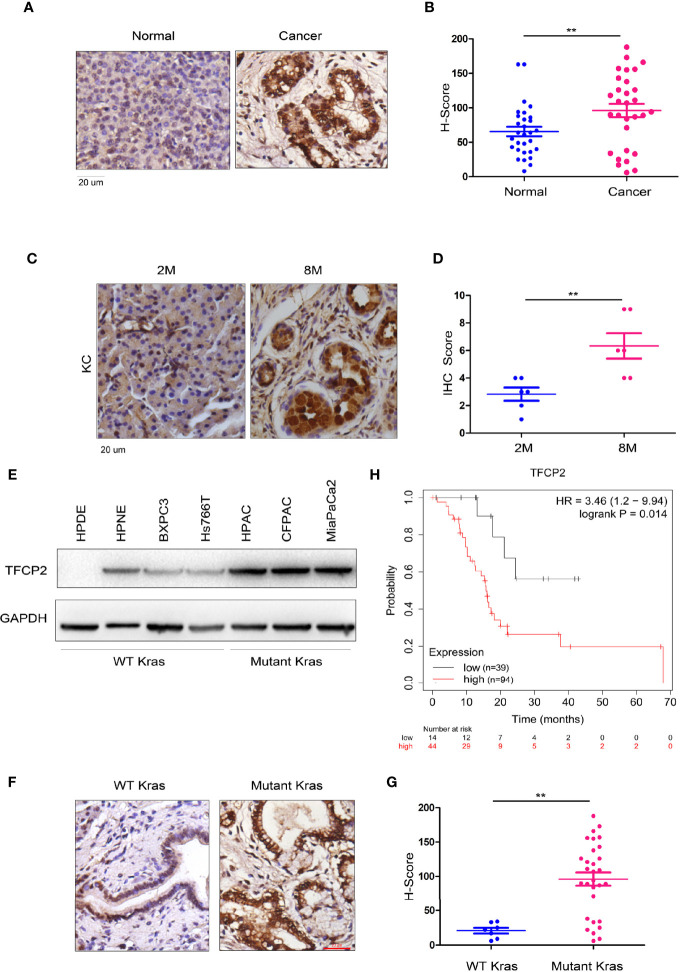
TFCP 2 was up-regulated in the progression of pancreatic cancer. **(A)** IHC was performed to examine the protein levels of TFCP2 in the clinical samples of pancreatic cancer and non-cancerous tissues (N = 32). **(B)** Statistical analysis was performed for **(A)**. **(C)** IHC was performed to examine the protein levels of TFCP2 in pancreatic tissues of KC mice with indicated age. **(D)** Statistical analysis was performed for **(C)**. **(E)** The protein levels of TFCP 2 in the normal pancreatic cell line (HPDE6C7 and HPNE) and cancer cell lines were examined using western blot. **(F, G)** IHC was performed to examine the protein levels of TFCP2 in the clinical samples of pancreatic cancer with (n = 25) or without (n = 7) Kras mutation (21 samples harboring G12D mutation, 1 samples harboring G12C mutation, 3 samples harboring G12V mutation), then the staining was scored and analyzed. **(H)** The survival analysis through mining the Kaplan-Meier Plot database **P < 0.01.

In order to further analyze the expression pattern of TFCP2 in pancreatic cancer, we used immunohistochemistry to determine the expression pattern of TFCP2 in pancreatic cancer. The results showed that the expression level of TFCP2 was upregulated in pancreatic cancer (**Figures 1A, B** and **Figure S1A**). Next, we detected the expression of TFCP2 in pancreatic tissues of KC (Pdx-Cre; Kras^G12D^) mice at different ages. Results showed that expression of TFCP2 was upregulated as malignancy progressed in KC mice (**Figures 1C, D** and **Figure S1B**). Moreover, the expression level of TFCP2 was higher in pancreatic cancer cells with KRAS mutation (**Figure 1E**). 90% patients with pancreatic ductal adenocarcinoma carried Kras mutation. We found that the expression level of TFCP2 was higher in pancreatic cancer tissues with Kras mutation (**Figures 1F, G** and **Figure S1C**). In addition, bioinformatics analysis using the Kaplan-Meier plotter database revealed that expression of TFCP2 negatively correlated with the survival (**Figure 1H**).

### TFCP2 Inhibited the Senescence of Pancreatic Cancer Cells

**Figure 2:**
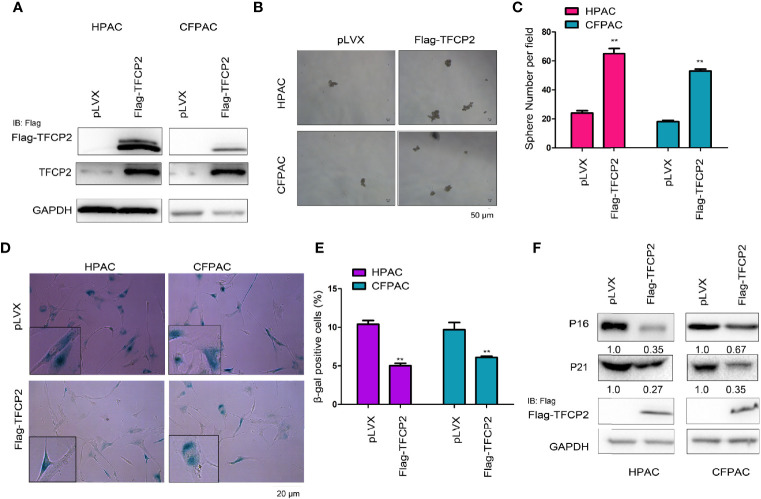
TFCP2 inhibited the senescence of pancreatic cancer cells. **(A)** The pancreatic cells were infected with the lentivirus for the overexpression of TFCP2. Cells were selected with puromycin and the resistant cells were pooled. The expression of exogenous and total TFCP2 was examined using western blot. **(B, C)** Sphere formation assay was performed to evaluate the effects of TFCP2 overexpression on the cell growth. **(D, E)** β-Gal staining was performed to evaluate the effects of TFCP 2 overexpression on the cell senescence. **(F)** The protein levels of senescence modulators were examined using western blot. The fold change was indicated. **P < 0.01.

In order to further understand the function of TFCP2 in pancreatic cancer, we overexpressed TFCP2 in pancreatic cancer cell lines (**Figure 2A**). Overexpression of TFCP2 promoted the formation of spheres (**Figures 2B, C**), and inhibited β-Gal staining (**Figures 2D, E**), suggesting that overexpression of TFCP2 possibly promoted the stemness and inhibited the senescence of pancreatic cancer cell. In agreement with this, senescence-related proteins, such as p16 and p21, were downregulated in TFCP2-overexpressed cells (**Figure 2F**).

**Figure 3:**
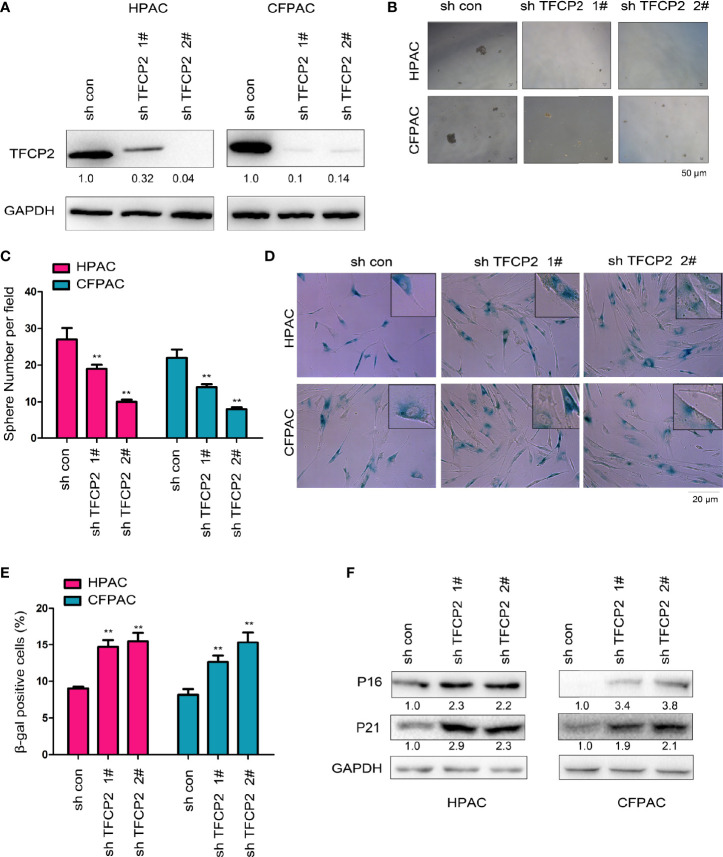
TFCP2 promoted the senescence of pancreatic cancer cells. **(A)** The pancreatic cells were infected with the lentivirus for the knockdown of TFCP2. Cells were selected with puromycin and the resistant cells were pooled. The expression of TFCP2 were examined using western blot. **(B, C)** Sphere formation assay was performed to evaluate the effects of TFCP2 knockdown on the cell growth, and the statistical analysis was performed. **(D, E)** β-Gal staining was performed to evaluate the effects of TFCP2 knockdown on the senescence, and the statistical analysis was performed. **(F)** The protein levels of senescence modulators were examined using western blot. The fold change was indicated. **P < 0.01.

Next, we interfered with the expression of TFCP2 in pancreatic cancer cells (**Figure 3A**). Interference with the expression of TFCP2 inhibited sphere formation (**Figures 3B, C**) and increased β-Gal staining signals (**Figures 3D, E**), suggesting that knockdown of TFCP2 could promote the senescence of pancreatic cancer cell. In agreement with this, senescence-related proteins such as p16 and p21 were upregulated in cells with the knockdown of TFCP2 (**Figure 3F**).

### Knocking Down of TFCP2 Induced Senescence in Cells With Constitutively Active Kras

**Figure 4:**
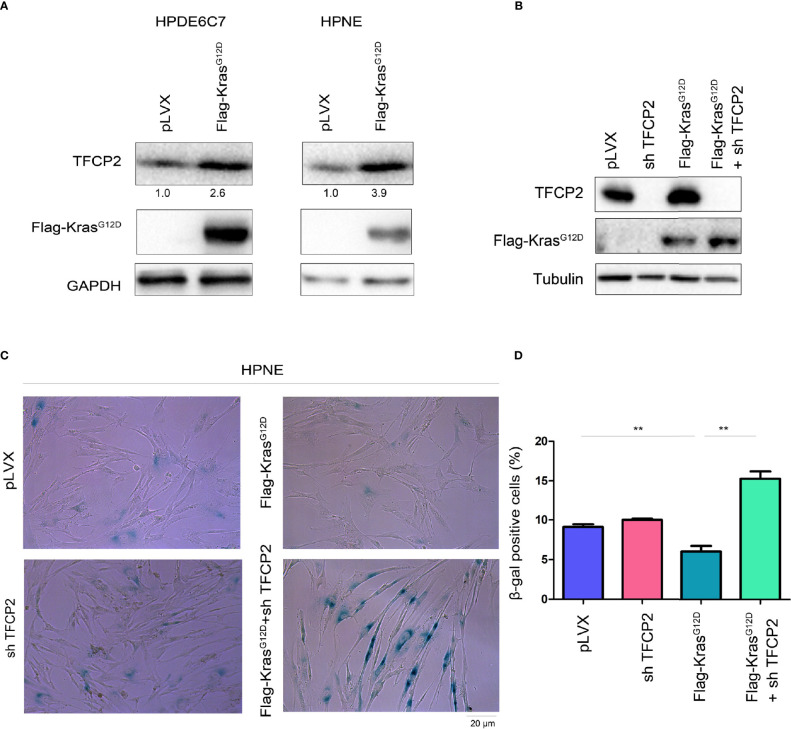
TFCP2 was essential for the Kras^G12D^ to bypass the senescence of pancreatic cancer cells. **(A)** Western blot was performed to examine the protein levels of TFCP2 in the pancreatic cancer cells. **(B)** Western blot was performed to examine the protein levels of TFCP2 and mutant Kras. **(C, D)** β-Gal staining was performed to examine the senescence. Statistical analysis was performed. **P < 0.01.

Constitutively active Kras (Kras^G12D^) can help tumor cells bypass senescence. We found that overexpression of constitutively active Kras in normal pancreatic cancer cells promoted the expression of TFCP2 (**Figure 4A**). We hypothesized that TFCP2 played a role in the process of constitutively active KRAS overcoming cell senescence. Therefore, we knocked down TFCP2 in HPNE cells with Kras^G12D^ overexpression (**Figure 4B**). Indeed, cell senescence occurred after expression of TFCP2 was knocked down (**Figures 4C, D**).

### TFCP2 Interacted With SREBP2 in Pancreatic Cancer Cells

**Figure 5:**
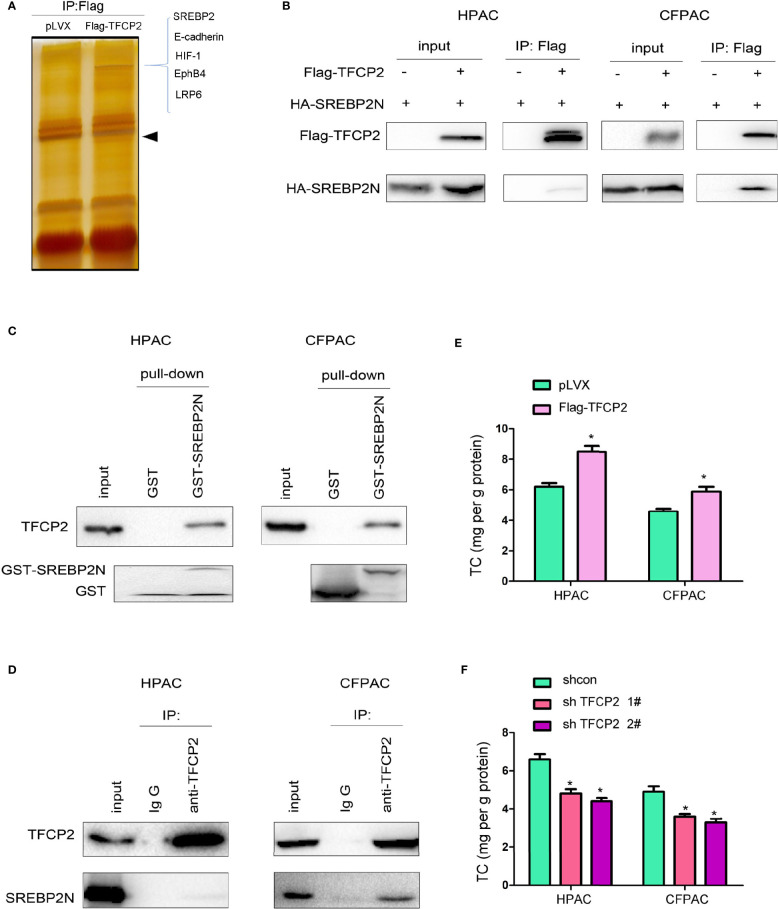
TFCP2 interacted with SREBP2. **(A)** The silver staining. HEK293T cells were transfected with the vector expressing TFCP2 (Flag-TFCP2). 48 hours later, cells were lysed and the immunoprecipitation was performed using anti-Flag antibody. The immunoprecipitates were separated with SDS-PAGE, and the gel was stained with silver. The differential band was cut for mass spectrum identification. Arrow head, the band for Flag-TFCP2. **(B)** Immunoprecipitation was performed 48 hours after cells were transfected with Flag-TFCP2 and HA-SREBP2(N) plasmids. **(C)** GST pull-down assay was performed after the cell lysate was incubated with the GST-SREBP2(N) fusion protein overnight. **(D)** Immunoprecipitation was performed to examine the interaction between endogenous TFCP2 and SREBP2(N). **(E, F)** The content of cholesterol in the cells with TFCP2 overexpression **(E)** or knockdown **(F)**. *P < 0.05.

In order to further reveal the mechanism underlying the inhibition of pancreatic cancer cell senescence by TFCP2, proteins interacted with TFCP2 were analyzed by mass spectrometry (MS) (**Figure 5A**). Among the candidate proteins was SREBP2, the regulator of cholesterol synthesis. It has been reported that SREBP2 can overcome senescence in glioma cells (23). We first verified the interaction between TFCP2 and SREBP2. The interaction between exogenously expressed TFCP2 and SREBP2 was detected in pancreatic cancer cells (**Figure 5B**). In addition, fusion protein GST-SREBP2N (1-485aa of SREBP2) could pull endogenously expressed TFCP2 from the cell lysate (**Figure 5C**). In the co-immunoprecipitation experiment, we could detect the interaction between endogenously expressed TFCP2 and SREBP2 (**Figure 5D**). Consistently, the content of cholesterol was increased in the HPAC and CFPAC cells with the overexpression of TFCP2 (**Figure 5E**), while the cholesterol was decreased when the expression of TFCP2 was knocked down (**Figure 5F**).

### Knockdown HMGCR or Treated the Pancreatic Cancer Cells With Statin Abolished the Inhibitory Effects of TFCP2 on Senescence

**Figure 6:**
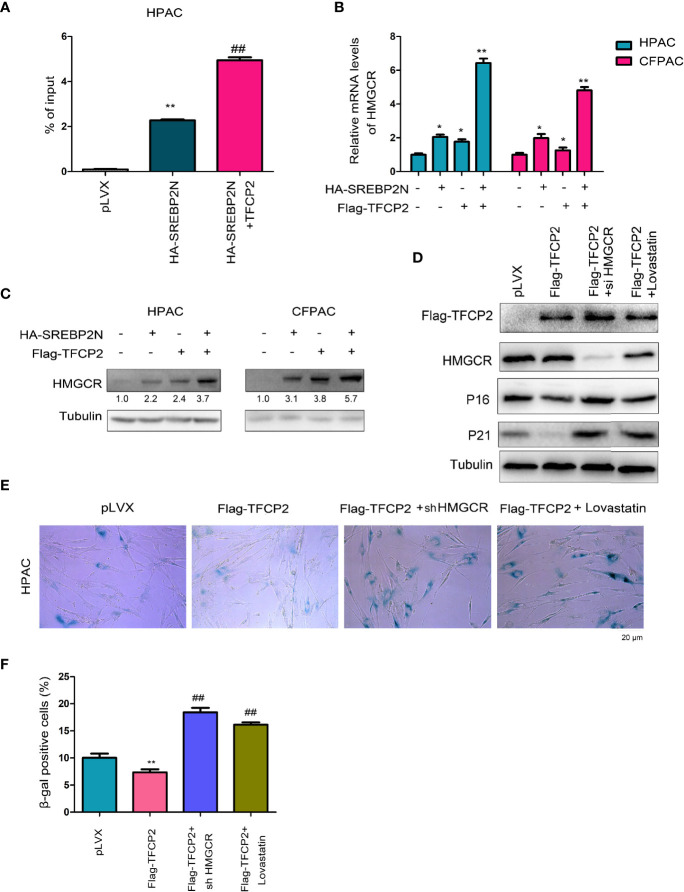
TFCP2 cooperated with SREBP2 to activated cholesterol synthesis. **(A)** ChIP assay was performed to examine the binding of SREBP2 with HMGCR promoter. ^##^ *P* < 0.01; **P < 0.01. **(B)** qPCR was performed to examine the mRNA levels of HMGCR. *P < 0.05; **, *P* < 0.01. **(C)** Western blot was performed to examine the protein levels of HMGCR. **(D)** Western blot was performed to examine the protein levels of P16, P21, TFCP2 and HMGCR. The concentration for Lovastatin was 20 µM. **(E)** β-Gal staining was performed to evaluate the senescence. The concentration for Lovastatin was 20 µM. **(F)** Statistical analysis for **(E)**. ^##^ *P* < 0.01; **P < 0.01.

HMGCR is one of the most important downstream target genes of SREBP2, and controls the cholesterol synthesis. The protein levels of SREBP2 and HMGCR were both up-regulated in pancreatic cancer tissues (**Figure S2**). In ChIP assay, we found that TFCP2 promoted SREBP2 binding to the promoter region of HMGCR (**Figure 6A**). At the expression level, TFCP2 and SREBP2 synergistically promoted the expression of HMGCR both at the mRNA levels and protein levels (**Figures 6B, C**). Next, HMGCR was knocked down in the HPAC cells with the overexpression of TFCP2, or the HPAC cells with the overexpression of TFCP2 were treated with statin (**Figure 6D**). It was found that the presence of statins treatment or interference with HMGCR promoted the senescence of cells with the overexpression of TFCP2, which was demonstrated by the staining of β-Gal and the expression of P16 and P21 (**Figures 6D–F**).

## Discussion

Pancreatic cancer is not sensitive to conventional radiotherapy, chemotherapy, and immunotherapy (24). Metabolic reprogramming is one of the most important characteristics of the pancreatic cancer (25). In our study, we investigated the effects of TFCP2 on the synthesis of cholesterol and the senescence of pancreatic cancer cell. The results suggest that inhibiting the function of TFCP2 and promoting the senescence of pancreatic cancer cell may be a new strategy for treating pancreatic cancer.

One of the most interesting findings of our study is that TFCP2 inhibits the senescence of pancreatic cancer cell. In our previous studies, we found that TFCP2 promoted the growth, colony formation, invasion and metastasis of pancreatic cancer cells (26). In this study, we found that TFCP2 inhibited the senescence of pancreatic cancer cell, further enriching the functions of TFCP2 in pancreatic cancer. Regulation of TFCP2 expression in liver cancer and expression of treatment-resistant and senescence-related gene have already been reported (27). Treatment of Hela cells with TFCP2 inhibitor FQI1 leads to polynuclear, apoptotic, and senescent Hela cells (22). These existing findings, plus our finding, further demonstrate the regulatory effect of TFCP2 on cell senescence.

Another interesting finding of our study is the regulation of cholesterol synthesis by TFCP2. What is known at present is that SREBP2 is an important regulatory transcription factor in cholesterol synthesis (28). However, it has been also reported that other proteins and SREBP2 can synergistically regulate cholesterol synthesis (28). For example, Deng et al. found that binding of β-catenin to the N-terminal of SREBP2 activated the expression of metabolic enzymes (such as HMGCR and IDI1) in the cholesterol synthesis pathway (29). Although functions of TFCP2 in tumors have been widely reported previously, their regulatory effects on the synthesis and metabolism of cholesterol are unknown. The regulation of cholesterol metabolism by TFCP2 that we revealed further highlights the important role of TFCP2 in tumor metabolism. The functions of TFCP2 in tumor metabolism are poorly understood so far. Further study to confirm the potential value of its inhibitor FQI1 in tumor therapy would be needed.

In conclusion, our study reveals the regulation of pancreatic cancer cell senescence and the underlying mechanism, and suggests that TFCP2 is a potential target for treating pancreatic cancer.

## Data Availability Statement

The raw data supporting the conclusions of this article will be made available by the authors, without undue reservation.

## Ethics Statement

The studies involving human participants were reviewed and approved by Ethics Committee of the Fudan University. The patients/participants provided their written informed consent to participate in this study. The animal study was reviewed and approved by Ethics Committee of the Fudan University.

## Author Contributions

DZ performed the IHC and senescence assay. PL performed the sphere formation assay. KZ performed the immunoprecipitation assay. HW analyzed the data and YD designed this study. All authors contributed to the article and approved the submitted version.

## Funding

This study was supported by grants from Shanghai municipal Health and Family Planning health Commission (201840036), JianFeng project of Shanghai Xuhui Commission of Health and Family Planning (SHXH201703) and Leading talent development fund of Minhang District, Shanghai.

## Conflict of Interest

The authors declare that the research was conducted in the absence of any commercial or financial relationships that could be construed as a potential conflict of interest.

## Publisher’s Note

All claims expressed in this article are solely those of the authors and do not necessarily represent those of their affiliated organizations, or those of the publisher, the editors and the reviewers. Any product that may be evaluated in this article, or claim that may be made by its manufacturer, is not guaranteed or endorsed by the publisher.

## Supplementary Material

Supplementary Figure 1 TFCP2 was up-regulated in pancreatic cancer. **(A)** Immunohistochemistry (IHC) was performed to examined the protein levels of TFCP2 in the pancreatic cancer tissues (cancer) and adjacent non-cancerous tissues (normal). **(B)** The protein levels of TFCP2 in the pancreatic tissues of KC mice aged 2 or 8 months. **(C)** IHC was performed to examined the protein levels of TFCP2 in the pancreatic cancer tissues with wide-type or mutant Kras. The magnification was 10-fold.

Supplementary Figure 2 SREBP2 and HMGCR was up-regulated in pancreatic cancer. Western blot was performed to examine the levels of SREBP2 and HMGCR protein in 6 pancreatic cancer tissues and paired non-cancerous tissues.
