# Supplementary figures and images for: TFCP2 Overcomes Senescence by Cooperating With SREBP2 to Activate Cholesterol Synthesis in Pancreatic Cancer
Source: Front Oncol. 2021 Nov 4;11:724437. doi: 10.3389/fonc.2021.724437 (PMC8599447; doi:10.3389/fonc.2021.724437)

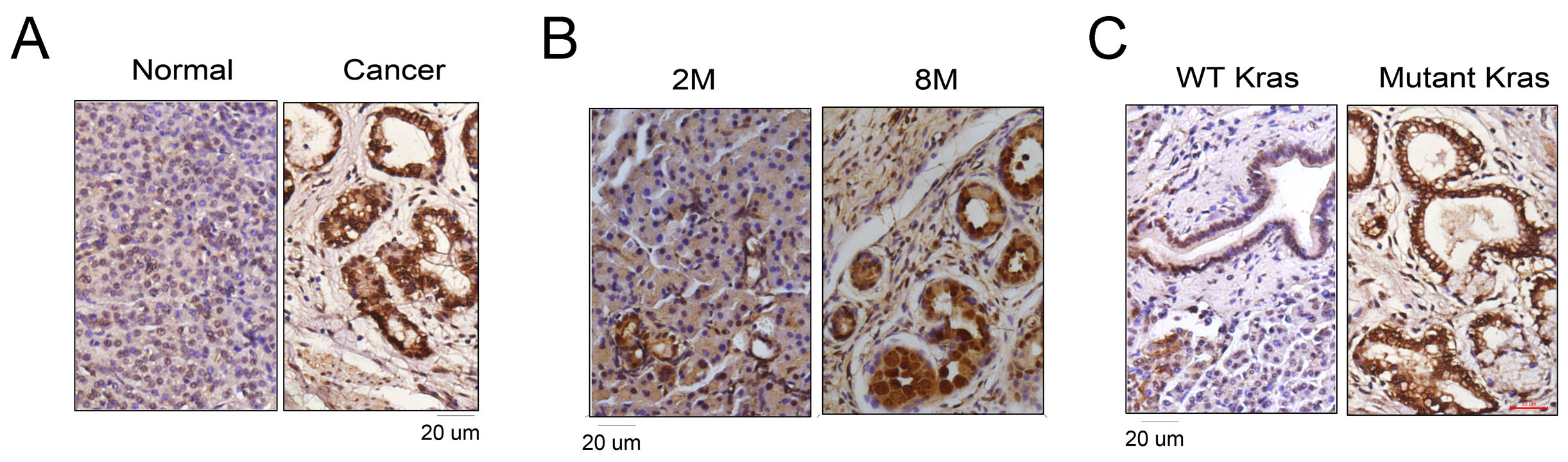

Supplement: Supplementary Figure 1 — TFCP2 was up-regulated in pancreatic cancer. (A) Immunohistochemistry (IHC) was performed to examined the protein levels of TFCP2 in the pancreatic cancer tissues (cancer) and adjacent non-cancerous tissues (normal). (B) The protein levels of TFCP2 in the pancreatic tissues of KC mice aged 2 or 8 months. (C) IHC was performed to examined the protein levels of TFCP2 in the pancreatic cancer tissues with wide-type or mutant Kras. The magnification was 10-fold. [file Image_1.jpeg]

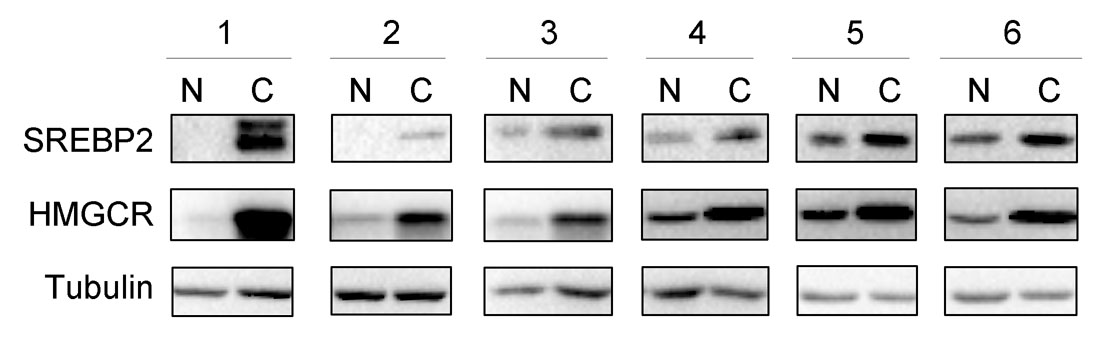

Supplement: Supplementary Figure 2 — SREBP2 and HMGCR was up-regulated in pancreatic cancer. Western blot was performed to examine the levels of SREBP2 and HMGCR protein in 6 pancreatic cancer tissues and paired non-cancerous tissues. [file Image_2.jpeg]
